# Sensitivity and specificity of PET/CT regarding the detection of lymph node metastases in prostate cancer recurrence

**DOI:** 10.1186/2193-1801-3-340

**Published:** 2014-07-04

**Authors:** Daniar K Osmonov, Diana Heimann, Isa Janßen, Alexey Aksenov, Almut Kalz, Klaus Peter Juenemann

**Affiliations:** Department of Urology and Pediatric Urology, University Hospital Schleswig Holstein, Campus Kiel, Arnold-Heller-Str. 7, 24105 Kiel, Germany

**Keywords:** Prostate cancer recurrence, Detection of lymph node metastases, PET CT scan

## Abstract

**Background:**

The aim of the study is to assess the efficacy of choline PET/CT regarding the detection of lymph node (LN) metastases in recurrent prostate cancer (PCa).

**Methods:**

49 patients with a biochemical recurrence of PCa (PSA >0.2 ng/ml) were included in the study. All patients were selected for further diagnostics with a choline-PET/CT. All patients underwent salvage extended lymphadenectomy. The PET/CT result and the histological findings were analyzed regarding the specificity and sensitivity and with respect to the localization of the metastases. The detection rate of LN metastases was analyzed with respect to interdependencies between the pre-PET/CT PSA-value as well as the role of prior ADT.

**Results:**

41 out of 49 (83.6%) patients showed positive PET/CT results. Positive LNs were found in 27 out of 49 patients (55.1%). 48.9% of the PET-CT-findings proved true positive, 36.7% were found to be false positive. 8.1% proved true negative and 8.1% false negative. This results in a specificity of 22.7% and a sensitivity of 85.1%. Out of the true positive PET/CT scans, 61.9% were not congruent regarding the localization of positive LNs. In patients with PSA [greater than or equal to] 5 ng/ml, the sensitivity of the PET/CT result was 93.7%, while specificity was 0%. In 24 patients who underwent ADT prior to the PET/CT diagnostics, the sensitivity was 84.6% and specificity 9.0%.

**Conclusions:**

The reliability of PET/CT imaging for detection of LN metastases is limited by a high false-positive rate. The influence of ADT further diminishes the PET/CT reliability. Sensitivity of the PET/CT is highest in patients with a PSA of [greater than or equal to] 5 ng/ml. Based on our results, we propose the following conclusions: 1. There is no well-established diagnostic alternative to Choline-PET/CT Scan. Therefore this method may continue to be performed in patients with BCR. 2. It is not sufficient to remove only those LNs that show up in the PET/CT. 3. Salvage extended lymphadenectomy should follow a predefined template (e.g. the “Kiel template”) and not just the PET/CT scan results.

## Background

In case of a post-treatment biochemical recurrence of prostate cancer (PCa), the diagnostic possibilities are currently limited to the distinction between a local recurrence and a systemic manifestation. By the most common definition, a biochemical recurrence after radical prostatectomy (RP) is present when the PSA-value reaches 0.2 ng/ml after RP (EAU Guidelines 2010; Pound et al. 1999). After radiation treatment (RT), a recurrence is present when the PSA is more than 2 ng/ml above the PSA nadir (EAU Guidelines 2010; Horwitz et al. 2005).

The application of the tracer 18F-FDG ([18F]-2Fluor 2desoxyglucose) in PET/CT is successfully used in many tumor types. However, a benefit in PCa diagnosis has been pulled into question by several authors, including Sanz et al. and Liu and co-workers (Sanz et al. 1999; Liu et al. 2001). There have been divergent results on Choline-PET/CT regarding PCa recurrence. The detection rates were analyzed and set in relation to the PSA level in several studies. While it was found to be a useful diagnostic method by Soyka and coworkers (Soyka et al. 2012), application of [11C]-Choline-PET/CT is only advisable when the PSA level is 1 ng/ml or higher according to Picchio et al. (2011). Other studies have shown the sensitivity of Choline-PET/CT to be as low as 41.1% (Scattoni et al. 2007; Schiavina et al. 2008). In Table 1, we have listed the results of different authors regarding sensitivity and specificity.

**Table 1:** **Comparison of PET/CT reliability based on the results from different authors**

	Sensitivity	Specificity	PPV	NPV
(Farsad et al. 2005)	66.0%	81.0%	87.0%	55.0%
(Castellucci et al. 2011)	93.0%	74.0%	60.0%	96.0%
(Schiavina et al. 2008)	41.4%	99.8%	94.4%	97.2%
(Budiharto et al. 2011)	9.4%	99.7%	75.0%	91.0%
(Kjölhede et al. 2013)	33.0%	92.0%	76.0%	65.0%

This study sets out to determine whether or not the method of PET/CT is suitable for accurate detection and localization of lymph node (LN) metastases. The radiological results are compared with the histological findings to validate the specificity and sensitivity of the method. In addition, our study aims to show whether or to which extent the rate of metastases detection may depend on the PSA level at the time of PET/CT-investigation. A further issue is the possible influence of androgen therapy on the PET/CT detection rate. Furthermore, the reliability of PET/CT in detecting skeletal metastases is compared with the reliability of bone scintigraphy. The crucial question is whether Choline-PET/CT should have an impact on the treatment decision in PCa recurrence. We have tried to formulate recommendations for clinical practice.

## Methods

This study is a retrospective analysis of case histories. The patients suffered a relapse of PCa, which was treated at our institution by salvage extended lymph node dissection (sELND) according to the “Kiel Template” between 2004 and 2012 (Osmonov et al. 2014).

49 patients (n = 49) were included in the study. Patients 1 to 10 previously underwent RT. Patients 11 to 49 (n = 39) underwent primary RP. 32 of these RP patients additionally underwent adjuvant RT. A biochemical relapse was observed in all patients and a Choline-PET/CT was performed in consequence. Those patients with primary RT underwent salvage prostatectomy (sRP) and sELND. The patients with primary RP underwent only sELND. The mean age of the patients was 65 years (range 52–76) at the time of salvage surgery.

18F-choline was applied in 43 patients, 11-choline was applied in 2 patients, 18FDG in one patient, and a SPECT/CT scan was conducted in 3 patients.

We also investigated the PET-CT true positive results concerning the localization of the metastases by distinguishing only roughly between the left and right side of the pelvis. Moreover, bone scintigraphy was performed in 38 patients. Thus it was possible to compare the current standard scintigraphy to the PET/CT result. The total PSA (tPSA) prior to PET/CT and salvage operation was analyzed to identify a possible impact on the PET/CT detection rate.

## Results

### PET/CT findings

In 8 of 49 cases there was an inconspicuous PET/CT in terms of LN metastases (16.3%). A positive PET/CT, indicating the presence of LN metastases, was found in 41 of 49 patients (83.6%).

### Histology

After removal of the pelvic LNs according to the “Kiel template” the pathological analysis, the result regarding the incidence of LN metastases was negative (pN0 status) in 22 cases (44.9%). The mean PSA level in these patients was 1.88 ng/ml. In 27 patients (55.1%), tumor cells were detected in one or more LNs. The mean PSA in these 27 patients was 8.76 ng/ml.

### Comparison of PET/CT and histological results

Table 2 shows the data distribution. In 46.9% of all cases the PET/CT was confirmed by the tissue examination and thus true-positive. In 36.7% of the cases, the positive PET/CT finding was not confirmed by the histological result, thus false-positive. In 8.2% of all cases, the negative PET/CT result was confirmed by histology and thus these were true false. In 8.2% of the cases, the negative PET/CT scan result was proven false-negative, as metastases were detected in the tissue samples.

**Table 2:** **Comparison of PET/CT results and histological analysis**

	Positive histology (27)	Negative histology (22)
**Positive PET/CT (41)**	23/49 (46.9%)	18/49 (36.7%)
**Negative PET/CT (8)**	4/49 (8.2%)	4/49 (8.2%)

### Specificity and sensitivity

There were 22 patients (44.9%) with a negative histology result (pN0). Out of these, 4 patients were also had a negative PET/CT result.

27 patients were found to have a positive histology result (55.1%). Out of these, 23 patients also had a positive PET/CT result.

The following formulas were used:

– Specificity = true-negatives / (true-negatives + false-positives)
– Sensitivity = true-positives / (true -positives + false-negatives)
– Positive predictive value (PPV) = true-positives / (true-positives + false-positives)
– Negative predictive value (NPV) = true-negatives / (true-negatives + false-negatives)

Thus the following results were calculated:

– Specificity = 4 / (18 + 4) = 18.2%
– Sensitivity = 23 / (23 + 4) = 85.2%
– PPV = 23 / (23 + 18) = 56.1%
– NPV = 4 / (4 + 4) = 50.0%

### Comparison of localization

For the true-positive results with a positive result both in imaging and histology (23/49), it is crucial to determine whether there is also a match of the detected LN metastasis localization. In the histology, 2 patients were found to have the metastases on the left and 4 on the right side of the pelvis, in 3 cases there were bilateral metastases. However, only in 9 out of the 23 true-positive cases (39.1%), the metastases were actually located where the PET/CT showed them. Thus the rate of true-positive results with correct localization was only 9 out of 49 (18.4%), with 32 false-positive results (65.3%), and the aforesaid 4 true-negative and 4 false-negative results (8.2%).

Under additional consideration of the correct localization, we calculated the following results regarding the specificity and sensitivity of the PET/CT:

- Specificity = 4 / (4 + 40) 9.1%
- Sensitivity = 9 / (9 + 4) = 69.2%
- PPV = 9 / (9 + 32) = 21.9%
- NPV = 4 / (4 + 4) = 50.0%

### Impact of the PSA value on the results

In the true-positive cases, the mean PSA level at the time of PET/CT and salvage surgery was 9.43 ng/ml and 2.08 ng/ml in the false-positive cases. In the true-negative cases, the PSA was 0.97 ng/ml and in false-negative cases it was 3.34 ng/ml.

We analysed the results by matching them with different PSA levels (≥5 ng/ml; ≥2- < 5 ng/ml; ≥1- < 2 ng/ml; <1 ng/ml) and compared them with the overall specificity (18.2%) and sensitivity (85.2%) as calculated for the entire cohort (see also Table 3).

**Table 3:** **Comparison of the PET/CT results at different PSA levels**

	PSA ≥5 ng/ml (n = 19)	PSA ≥2- < 5 ng/ml (n = 8)	PSA ≥1- < 2 ng/ml (n = 12)	PSA <1 ng/ml (n = 10)
True-positive	15/19 (78.9%)	4/8 (50.0%)	4/12 (33.3%)	0
False-positive	3/19 (15.8%)	2/8 (25.0%)	6/12 (50.0%)	7/10 (70.0%)
True-negative	0	0	2/12 (16.7%)	2/10 (20.0%)
False-negative	1/19 (5.3%)	2/8 (25.0%)	0	1/10 (10.0%)
Specificity	-	-	25.0%	22.2%
Sensitivity	93.8%	66.7%	100.0%	-

PSA ≥5 ng/ml (mean: 11.89 ng/ml), n = 19: the PET/CT was true-negative in 0, true-positive in 15, false-positive in 3 and false-negative in 1. Therefore, the sensitivity is 93.8%.

PSA ≥2- < 5 ng/ml (mean: 3.49 ng/ml), n = 8: of these, the PET/CT was true-negative in 0, true-positive in 4, false-positive in 2 and false-negative in 2. Therefore, the specificity is 0% and the sensitivity is 66.6%.

PSA ≥1- < 2 ng/ml (mean: 1.51 ng/ml), n = 12: of these, the PET/CT was true-negative in 2, true-positive in 4, false-positive in 6 and false-negative in 0. Therefore, the specificity is 25% and the sensitivity is 100%.

PSA <1 ng/ml (mean: 0.68 ng/ml), n = 10: of these, the PET/CT was true-negative in 2, true-positive in 0, false-positive 7 and false-negative in 1 patient. Therefore, the specificity is 22.2% and sensitivity 0%.

### Impact of ADT

A total of 24 out of 49 patients underwent ADT. We compared the patients who were treated with ADT (until maximum 4 weeks prior to PET/CT) with those who were not. We registered only slight differences between the two groups (Table 4). However, the false-negative results seem to be somewhat higher without ADT. When comparing the specificity, it appears that there is no difference (both 9.1%), while sensitivity is slightly higher (84.6% and 78.0%) under ADT.

**Table 4:** **Comparison of the PET/CT results of patients with and without previous ADT**

	***With***hormonal therapy (N = 24)	***Without***hormonal therapy (N = 25)
**True-positive**	11/24 (45.8%)	11/25 (44.0%)
**False-positive**	10/24 (41.7%)	10/25 (40.0%)
**True-negative**	1/24 (4.2%)	1/25 (4.0%)
**False-negative**	2/24 (8.3%)	3/25 (12.0%)
**Specificity**	(9.1%)	(9.1%)
**Sensitivity**	(84.6%)	(78.6%)

### Impact of the Gleason score

Poorly differentiated tumors with Gleason scores of 8–10 are present in 21 of 49 cases. The specificity of PET/CT metastases detection in this group is 22.2% and sensitivity 83.3%. A preoperative Gleason-Score of 7 is present in 18 patients with a specificity of 33.3% and sensitivity of 83.3%. Gleason-Scores of 5–6 are present in 6 cases, a sensitivity of 33.3% and a not calculable specificity. For the two patients with Gleason-Scores of 2–4, neither specificity nor sensitivity was calculable.

### Correlation of PET/CT and bone scintigraphy

38 patients of the 49 patients additionally underwent a bone scan. Of these, 8 scintigraphy images led to diagnosis of bone metastases (21.1%). In 2 of the 38 patients the suspicion of bone metastases was based on the PET/CT. A correlation of the bone scintigraphy and PET/CT results is shown in Table 5. The PET/CT detection capability of bone metastases showed a specificity of 96.7%, a sensitivity of 12.5%, a PPV of 50.0% and a NPV of 80.6%.

**Table 5:** **Comparison of bone scintigraphy and PET/CT regarding bone metastases**

	Bone scintigraphy positive (8)	Bone scintigraphy negative (30)
**PET/CT positive (2)**	1/2 (50.0%)	1/2 (50.0%)
**PET/CT negative (36)**	7/36 (19.4%)	29/36 (80.6%)

## Discussion

At present, the EAU guidelines reject a recommendation of the PET/CT due to the lack of evidence (EAU Guidelines 2010). Nevertheless, Scattoni et al. report high detection rates, even for patients with low PSA-levels, while conceding that the detection rate is limited at low PSA-levels due to the non-discovery of microscopic metastases (Giovacchini et al. 2012). Furthermore, Schiavina and et al. argue that although a PET/CT sensitivity of 60% is low in terms of LN metastases detection, it is still higher than clinical nomograms. This study group reports a specificity of 100% (Schiavina et al. 2008). A further study by Soyka et al. shows that the results of a PET/CT examination can lead to a change in the treatment plan; thus the treatment strategy was changed from curative to palliative and or from palliative to curative due to the imaging results in 14% of the patients (Soyka et al. 2012).

On the other hand, Rybalov et al. argue that the accuracy of metastases detection is too small to justify the application of Choline-PET/CT as a standard screening test for patients with recurrent PCa (Rybalov et al. 2012). Moreover, Salminen et al. point out that negative PET/CT results should be judged carefully in particular (Salminen et al. 2002). The DGU (Deutsche Gesellschaft für Urologie - German Society of Urology) suggest that neither PET/CT nor other imaging methods should be used as an alternative to biopsy (Wirth et al. 2009). Furthermore, Murphy et al. have put forward that incorrect results might be reduced by training radiologists to distinguish more effectively between a normal and pathological distribution of PET tracers (Murphy et al. 2011). Budiharto et al. found even lower results when analyzing the histology after sELND (see Table 1) (Budiharto et al. 2011).

Ultimately, histological examination remains the gold standard in the investigation of potentially malignant tissue. Despite a negative PET/CT result, tumorous tissue may already be present in the LNs, as metastases are often too small to show up in the image.

In contrast to the current analysis, nearly all former studies on PET/CT reliability for LN metastases detection were restricted to patients with positive PET-CT results. Moreover, subsequent lymphadenectomy is often performed as limited PLND or semi-extended PLND. In the current study we performed sELND according to the “Kiel template” and the “Kiel salvage surgical principles” (Osmonov et al. 2014). The “Kiel template” includes the following anatomic regions: (1) para-aortal, (2) interiliacal, i.e. the area between the right and left common iliac artery, (3) both sides of the common iliac artery, (4) the region around the promontorium, (5) the presciatic area resp. the “triangle Marcille”, (6) the region of the internal iliac artery, (7) the fossa obturatoria, (8) the region of the external iliac artery, and (9) the sacral region (Osmonov et al. 2014).

In the “Kiel salvage surgical principles”, we choose a transperitoneal access and define landmarks such as the iliac vessels before beginning with the dissection. The ureter is identified and separated carefully from the surrounding tissue. LN dissection is then performed systematically from top downwards. Small or medium clips are used to avoid extensive ligation. Moreover, we use the harmonic scalpel to seal the LN vessels and to shorten the operation time (Osmonov et al. 2014).

Despite thorough dissection of all relevant lymph nodes, we found a high percentage of patients with false-positive PET/CT results (36.7%). Although 46.9% of the patients were found to be true-positive for metastases, it can be concluded that the PET/CT tends to show a positive rather than a negative result, with a high rate of false-positives.

Furthermore the high rate of false-negative results in the PET/CT should also be considered carefully. In 4 out of 8 PET/CT-negative patients, the result proved false-negative, - in other words, these patients had histologically proven LN metastases (8.2% of all patients). These patients would have been declared as LN metastases-free, although they were not, and thus they would have been treated inadequately resp. undertreated if the treatment decision had been made on the PET/CT alone.

Based on our data, we calculated a PET/CT specificity of 18.2% and a sensitivity of 85.2%. A data distribution with much higher sensitivity than specificity generally shows an overestimation of positive results, i.e. the number of those patients who were mistakenly diagnosed with LN metastases. The rate of patients, whose PET/CT finding was confirmed by histology, was even lower. The predictive values of the test were both very low (PPV: 56.0% and NPV 50.0%).

When considering the lack of the PET/CT capacity to show the correct localization of the lesions, the specificity and sensitivity drop even further (9.1% and 69.2%). In the current study, the only location criteria were left or right side of the pelvis, therefore the accuracy of the localization has been simplified. Despite this, there is no match in terms of localization in 14 out of 23 (60.9%) of the true-positive findings in the PET/CT. Consequently, the localization of metastases in the PET/CT was actually confirmed by pathological examination in only 9 out of all 49 patients (18.4%), or, in relation to all positive PET/CT findings, 9 of 41 (21.9%). This proportion seems too small to base the treatment decision on the PET/CT alone. Even if surgical therapy was correctly chosen due to the PET/CT, the ensuing surgery might be applied in the wrong location.

Various publications show a lower detection capability of the PET/CT in PCa recurrences with small PSA values. Schillaci et al. recommend its use in patients with PSA >2 ng/ml. According to their studies, the detection rates are 20% at a PSA-value of 1 ng/ml, 55% at 1- ≤ 2 ng/ml, 80% at 2- ≤ 4 ng/ml and 87% at >4 ng/ml (Schillaci et al. 2012). These results are similar to the findings of the current study. Graute et al. found that the PET/CT has a relatively high detection capability (specificity 74% and sensitivity 82%) above a PSA cut-off value of 1.74 ng/ml (Graute et al. 2012).

Although it seems logical that patients with histologically proven LN metastases have a much higher mean PSA compared to those with negative tissue samples (8.76 ng/ml vs 1.88 ng/ml), our study did not clearly confirm the dependence of true-positive PET/CT results on the PSA level. The highest detection rates occurred in the PSA range of ≥1- < 2 ng/ml, with a sensitivity of 100% but a specificity of only 25%. In summary, it can be assumed that PET/CT imaging overestimates the percentage of LN metastases in patients with low PSA values ≥1.

Moreover, we have also not been able to confirm a clear impact of ADT prior to PET/CT, which is often put forward as a limiting factor in the literature. For example, Fuccio et al. found that ADT preceding PET/CT significantly decreases the detection capability due to a lower choline uptake in tumorous cells when androgen has been deprived (Fuccio et al. 2011). Based on our current study, we conclude that hormonal treatment has no effect on the PET/CT detection ability. Sensitivity and specificity rates are comparable in fact, with a slightly higher sensitivity in those patients with previous ADT.

When looking at the correlation with Gleason Score, we found the highest specificity (33.3%) at GS 7 and a relatively high sensitivity at 83.3% in patients with Gleason Scores 7 and above. However, in the group GS 8–10, the PET/CT missed metastases in 2 out of 21 patients (11.1%). There was a true-positive result in 55.5%, so the value is only somewhat higher than the overall average (46.9%). Overall it seems that while Gleason scores of or above 7 slightly increase the PET/CT’s sensitivity, specificity remains low throughout.

In addition, we examined the capability of PET/CT regarding the detection of bone metastases. The gold standard of bone scintigraphy, has sensitivity and specificity rates of up to 100%, as reported by Even-Sapir (Even-Sapir et al. 2006). In another study, Picchino et al. found that in PET/CT the specificity (98–100%) was higher, but the sensitivity (89%) was lower than in bone scintigraphy; thus they conclude that PET/CT cannot replace bone scintigraphy, while claiming that a positive result for bone metastases in the PET/CT is relatively reliable (Picchio et al. 2012).

In our study, by contrast, we found a true-positive rate of only 50.0% and a high false-negative rate (19.4%) when comparing PET/CT with scintigraphy. Therefore, we believe that PET/CT should not be used for distinct diagnosis.

## Conclusion

We found neither as clear influence of the Gleason-Score nor of any specific PSA range on the reliability of the PET/CT result, even though some authors claim that the PSA value makes a big difference. While controversial views persist, the actual impact remains uncertain at this point. Furthermore, it was also not possible to verify any influence of ADT on the accuracy of LN detection with PET/CT imaging. Moreover, bone scintigraphy is the gold standard and PET/CT cannot be given the same relevance.

It is important to emphasize that it is risky to base a treatment plan on the results of the PET/CT only as false-positive and false negative findings may lead to inadequate treatment, especially when including the inadequate localization of LN metastases in the consideration.

Unfortunately, there is no reliable diagnostic method for staging an advanced PCa respectively LN metastasis in patients with BCR. The challenge for the future is to identify a more reliable imaging method for LN metastases detection, either by PET/CT with a more specific tracer or by a completely different method.

To deal with the problem in the present situation we propose adherence to “three Kiel principles” which are based on our experience in PCa relapse salvage surgery:

1. There is no a well-established diagnostic alternative to Choline-PET/CT Scan. Therefore this method may still be performed in patients with BCR. But as soon as a valid new diagnostic method becomes available, it should be considered carefully as a possible replacement of PET/CT.
2. It is not sufficient to remove only the LNs from the regions with positive PET/CT imaging results. Our analysis of sensitivity and specificity in relation to the localization showed a low specificity value of 9.1% and a sensitivity of only 69.2%.
3. LN surgery should follow a predefined template (for example the “Kiel template”) and not only the PET/CT scan results (Pound et al. 1999) due to the risk false negative results in the PET/CT.

This study was approved by the appropriate Ethics Committee (Ethics Committee of the University Hospital Schleswig Holstein) and thus performed in accordance with the ethical standards laid down in the 1964 Declaration of Helsinki and its later amendments.

All persons gave their informed consent prior to inclusion in the study. Details that might disclose the identity of the subjects under study have been omitted.

### Ethical approval

Current study has been performed in accordance with the ethical standards. Statistical evaluations were done anonymously and details that might disclose the identity of the subjects under study were omitted. Current manuscript represents a retrospective evaluation and analysis of our prospective and consecutive salvage lymph node’s data bank, which was done by a separate hospital unit (third party).
